# Strengthening monkeypox diagnostics and healthcare amidst Covid-19 realities: A call to action

**DOI:** 10.1016/j.amsu.2022.104898

**Published:** 2022-11-13

**Authors:** Ernesto Oluwafemi Dibia, Damilola Quazeem Olaoye

**Affiliations:** University of Ibadan, Nigeria; 68 Nigerian Army Reference Hospital, Yaba, Lagos, Nigeria; University of Ibadan, Nigeria; Lakeshore Cancer Center, Victoria Island, Lagos, Nigeria

**Keywords:** Monkeypox virus, Healthcare systems, Covid-19, Public health

## Abstract

Monkeypox virus is a zoonotic DNA virus related to the virus that causes smallpox. It was first isolated and identified in 1958 but its first confirmed human case was in 1970 when the virus was isolated from a child in the Democratic Republic of Congo. Since then, several cases have been reported within the African continent and globally. Despite its spread, Monkey pox disease has continued to suffer neglect in terms of research and funding due to its reported inefficiency in transmitting from Man to man as its transmission was reportedly limited to its endemic regions in Africa. Poor health data management, inadequate capacities in terms of testing infrastructure and health care workers and weak surveillance systems are some of the challenges faced by African countries. Multisectoral collaboration in breaking the transmission cycle of monkeypox infection and other preventive measures must be improved upon. Community advocacies and education play integral roles in infection spread preparedness, as well as in strengthening the healthcare system.

## Highlights

• Monkeypox virus infection remains a challenging health threat in sub-Saharan Africa, and the world at large.
• Research, monitoring, supporting, and sharing information among countries affected by monkeypox should be improved.
• Research priorities towards monkeypox virus vaccine production should be prioritised.
• Sensitization toward the populations at risk must be invested into.

## 1 Introduction

Monkeypox virus, as the name implies, is a zoonotic DNA virus related to the virus that causes smallpox, belonging to the Orthopoxvirus genus of the Poxviridae family. While it was first isolated and identified in 1958 [1], its first confirmed human case was in 1970 when the virus was isolated from a child in the Democratic Republic of Congo [2]. Since then, several cases reported within the African continent have continued to originate from initial contact with wildlife reservoirs [3]. The definitive animal reservoir of MPXV has not been ascertained. Monkeypox disease has continued to suffer neglect in terms of research and funding due to its reported inefficiency in transmitting from Man to man as its transmission was reportedly limited to its endemic regions in Africa [4,5]. Animal-to-human can occur through handling and processing infected animals products. Bites and scratches from these infected animals may also result in transmission. Transmission of monkeypox virus occurs through large respiratory droplets, close or direct contact with skin lesions, and possibly through contaminated fomites [6]. There has been no clear evidence of sexual transmission although vertical transmission and fetal deaths have been well reported [7].

Furthermore, several studies have reported coextensive immunity to monkeypox virus through vaccination against smallpox. However, eradicating smallpox and subsequent lack of vaccination efforts have paved the way for monkeypox to gain clinical relevance [8]. Also, there is a high possibility of underreporting due to the fact that most cases of monkeypox occur in rural Africa, which may lead to direct underestimation of the potential threat to public health [9].

However, more than 3000 monkeypox virus infections have been reported in over 50 countries across five regions, since May 2022. This has prompted the World Health Organization to declare monkeypox an “evolving threat of moderate public health concern” [10].

With an increasing number of cases being reported across regions, it is important to update knowledge of this re-emerging Public health threat, including its diagnosis prevention and clinical management in a bid to prepare health systems for the present reality.

## 2 Monkeypox prevention

Monkeypox virus is a viral zoonotic disease, that chiefly occurs in regions of central and Western Africa, while being exported occasionally to other parts of the world. It can be classified under the genus orthopoxviruses in the family Poxviridae, and is a double-stranded DNA virus according to the Baltimore classification of viruses [11] Like most other poxviruses, monkeypox virus is transmitted to human beings during interactions between man and infected animals. Having its first isolation from monkeys, the monkeypox virus thrives in other natural hosts like the Gambian pouched rats, dormice, tree squirrels, and rope squirrels [12].

For many viral infections, vaccination is a great measure of prevention. Seeing as monkeypox virus has many genetic similarities with smallpox, data further suggest that vaccination against smallpox infection is believed to confer about 85% cross-protection against monkeypox, and may improve the clinical manifestation of infected individuals [13]. However, regarding people in close contact with an infected patient, prevention of monkeypox can prove to be very difficult. This is because of the ease of viral transmission via direct contact with skin lesions and through infected items [12]. Hence, some of the best measures in preventing monkeypox infection include:

● Isolation and euthanization of animals who have been suspected to be the reservoirs of the monkeypox virus [14].
● Isolation of infected patients in a negative pressure room to prevent any form of human-to-human outflow and spread of the virus [15].
● Eliminating contact with infected material that may have been in contact with the infected human or animal
● Use of proper protective equipment for front-line medical workers when managing infected patients and other high-risk individuals [16].
● Avoiding direct contact with infected individuals, animals, and suspected animals of monkeypox virus in geographical regions having high monkeypox prevalence [17].

## 3 Monkeypox diagnostics and treatment

Clinical and epidemiological data must be correlated with diagnostic assays in order to confirm monkeypox infection. This data may include the date of onset of fever, date of rash onset, current state of individual, as well as age. The data collection helps in the identification of people that have a higher risk of false positive results (recently vaccinated individuals with a vaccinia-based vaccine).

The confirmation of monkeypox infection is based on the sum total of patient history, clinical presentations, and confirmatory laboratory testing - these tests include Polymerase chain reaction (PCR), Enzyme-linked immunosorbent assay (ELISA), Immunohistochemistry, and Western blot [17].

Isolation of the viral nucleic acid is also done using lesion exudates, dry crusts, and biopsy to isolate further and confirm the diagnosis. This confirmatory diagnosis plays a critical role in eliminating the possibility of other possible infections like smallpox [18]. While Western blot analysis on monkeypox virus proteins is used to identify and confirm monkeypox viral infection, the World Health Organization recommends RT-PCR test for diagnosis and confirmation due to its accuracy and sensitivity [19].

In most cases, monkeypox usually presents with mild to moderate symptoms, and patients may not necessarily need therapy for recovery. There are no specific treatments for monkeypox virus infections, however, symptom alleviation, complication management, and prevention of long-term sequelae should be optimal during the clinical care for monkeypox infection [19].

For specific care, an antiviral, tecovirimat, which was developed for smallpox, has been approved for the treatment of monkeypox in 2022, based on recovery data derived from animal and human clinical studies. Although it is not widely available yet, if necessary, tecovirimat must be used under excellent clinical supervision [14].

## 4 Current realities and prospects

Much is still yet to be known regarding monkeypox virus. However, as of June 22, 2022, only one death out of 3413 confirmed cases of monkeypox has been recorded. Various public health agencies like the World Health Organization and Centre for Disease Control (CDC) have played critical role in monitoring, supporting, and sharing information of monkeypox virus among member states. The overall risk of monkeypox virus globally has been determined to be moderate, according to the resolution of the International Health Regulations Emergency Committee in June 2023. Because of the existing concurrent covid-19 health precautions, case finding, contact tracing, laboratory investigations, and various forms of clinical management have been more seamless in the control of monkeypox infection.

While genomic sequencing of the monkeypox virus DNA is still ongoing, immunization against monkeypox is encouraged. Since the 1980s, the smallpox vaccination, which offer some protection against monkeypox, has been discontinued, leaving a huge portion of the population exposed to the monkeypox virus. In recent years, only a very limited number of military personnel, frontline medical personnel, and laboratory personnel have received the smallpox vaccine, and still possess immunity against monkeypox.

With the world just recovering from the global Covid 19 Pandemic, there has been some comparison between the MPXV and the Coronavirus 19. However, unlike coronavirus 19, the MPVX is not a novel virus as there are current experienced expertise in prevention and management gathered from previous outbreaks. The challenges in Africa regarding containment of Monkey Pox disease is quite similar to that encountered during Covid 19 outbreak. The challenges faced by Africa include poor health data management, inadequate capacities in terms of testing infrastructure and health care workers complexed with the fact that the surveillance systems in many African countries are weak.

Several studies in sub-Saharan Africa identified weaknesses and deficiencies in the quality of data collected for the surveillance of infectious diseases. The reported deficiencies have ranged from incomplete data to inconsistent data at different levels of the surveillance system. Without a doubt, several factors have contributed to the continuous persistence of these reported weaknesses. They include but are not limited to low motivation, inadequate health management information systems–related resources, and lack of skills among healthcare workers owing to the lack of training.

A lack of MPX knowledge among health care workers and communities will also increase cases of underreporting, increased spread and improper management of cases, and poor prevention and control strategies, leading to the further spread of the disease.

If the past outbreaks of infectious diseases in Africa are anything to go by, there's an urgent need to strengthen Africa's preparedness in tackling these issues. These strengthening need to bring together concerted efforts targeted at increasing research funding to bridge gaps in knowledge and introduce new perspectives to current issues. Strengthening of surveillance systems should be done through active, real-time digitalization of health care data with secure data sharing for research and policy decisions. The place of multisectoral collaboration can not be under estimated in controlling the current realities. There is an urgent need for collaboration with the veterinary communities in understanding the disease reservoir and trends of transmission. Community advocacies should be geared towards increasing preparedness and the need for active preventive measures. High-risk individuals such as sex workers, immunocompromised individuals, children and other key populations should be well-sensitized about risks and prevention.

## 5 Conclusion

As newer cases unfold, we will gain new perspectives to the impending threats posed by the Monkeypox virus. Africa as a continent, needs to act fast and effectively in curbing the spread and casualties from the infection spread. Although there have been a lot of challenges inherent to Africa, lessons learned from previous outbreaks need to be applied in winning the current fight against monkeypox infection, and preventing another full-blown pandemic.

## Ethical approval

No ethical approval was required or obtained for this study.

## Sources of funding

This study was self-funded

## Author contribution

This review was conceptualized by Damilola Quazeem Olaoye and Ernesto Dibia. Ernesto Oluwafemi Dibia and Damilola Quazeem Olaoye contributed to writing the manuscript. All the authors read and approved the final manuscript.

## Conflicts of interest

There are no conflicts of interest.

## Trial registry number

1. Name of the registry:
2. Unique Identifying number or registration ID:
3. Hyperlink to your specific registration (must be publicly accessible and will be checked):

## Guarantor

DIBIA, Ernesto Oluwafemi.

OLAOYE, Oluwadamilola Quazeem.

## Consent

Not applicable.

## Availability of data and materials

Not Applicable.
